# A Critical Role of TRPM7 As an Ion Channel Protein in Mediating the Mineralization of the Craniofacial Hard Tissues

**DOI:** 10.3389/fphys.2016.00258

**Published:** 2016-07-06

**Authors:** Yukiko Nakano, Michael H. Le, Dawud Abduweli, Sunita P. Ho, Lillia V. Ryazanova, Zhixian Hu, Alexey G. Ryazanov, Pamela K. Den Besten, Yan Zhang

**Affiliations:** ^1^Department of Orofacial Sciences, University of California, San FranciscoSan Francisco, CA, USA; ^2^Center for Children's Oral Health Research, University of California, San FranciscoSan Francisco, CA, USA; ^3^Preventive and Restorative Dental Sciences, University of California, San FranciscoSan Francisco, CA, USA; ^4^Department of Pharmacology, Robert Wood Johnson Medical SchoolPiscataway, NJ, USA

**Keywords:** TRPM7, enamel, dentin, bone, ion transport, biomineralization, alkaline phosphatase, magnesium homeostasis

## Abstract

Magnesium ion (Mg^2+^) is the fourth most common cation in the human body, and has a crucial role in many physiological functions. Mg^2+^ homeostasis is an important contributor to bone development, however, its roles in the development of dental mineralized tissues have not yet been well known. We identified that transient receptor potential cation channel, subfamily M, member 7 (TRPM7), was significantly upregulated in the mature ameloblasts as compared to other ameloblasts through our whole transcript microarray analyses of the ameloblasts. TRPM7, an ion channel for divalent metal cations with an intrinsic serine/threonine protein kinase activity, has been characterized as a key regulator of whole body Mg^2+^ homeostasis. Semi-quantitative PCR and immunostaining for TRMP7 confirmed its upregulation during the maturation stage of enamel formation, at which ameloblasts direct rapid mineralization of the enamel matrix. The significantly hypomineralized craniofacial structures, including incisors, molars, and cranial bones were demonstrated by microCT analysis, von Kossa and trichrome staining in *Trpm7*^Δkinase∕+^ mice. A previously generated heterozygous mouse model with the deletion of the TRPM7 kinase domain. Interestingly, the skeletal phenotype of *Trpm7*^Δkinase∕+^ mice resembled those found in the tissue-nonspecific alkaline phosphatase (*Alpl*) KO mice, thus we further examined whether ALPL protein content and alkaline phosphatase (ALPase) activity in ameloblasts, odontoblasts and osteoblasts were affected in those mice. While ALPL protein in *Trpm7*^Δkinase∕+^ mice remained at the similar level as that in *wt* mice, ALPase activities in the *Trpm7*^Δkinase∕+^ mice were almost nonexistent. Supplemented magnesium successfully rescued the activities of ALPase in ameloblasts, odontoblasts and osteoblasts of *Trpm7*^Δkinase∕+^ mice. These results suggested that TRPM7 is essential for mineralization of enamel as well as dentin and bone by providing sufficient Mg^2+^ for the ALPL activity, underlining the key importance of ALPL for biomineralization.

## Introduction

Magnesium is the fourth most common cation in the human body, and is the second most abundant cellular cation (Romani, 2011). Intracellularly, by binding to the enzymes, magnesium functions as an essential activator of enzymes (Cowan, 2002; Maguire and Cowan, 2002; Sreedhara and Cowan, 2002), and by binding to nucleic acids, it contributes to the second messenger systems and modification of nucleic acid structure (Neitzel et al., 1991; Barciszewska et al., 2001). Moreover, it binds to cellular membrane components, including ion channels, and affects fluidity and permeability of molecules (Wolf and Cittadini, 2003; Wolf et al., 2003). Magnesium deficiency in humans is known to be associated with skeletal diseases, including hypocalcemia and osteoporosis, due to impaired parathyroid hormone (PTH) secretion, renal and skeletal resistance to PTH and vitamin D, and increasing inflammatory cytokines, like interleukin (IL) -1 and tumor necrosis factor (TNF) -α (Weglicki et al., 1996; Rude and Gruber, 2004; Rude et al., 2004; Rude and Shils, 2006). In animal models treated with low Mg^2+^ diet, dentin and enamel mineralization defects are reported (Irving, 1940; Bernick and Hungerford, 1965; Trowbridge et al., 1971).

Transient receptor potential melastatin-subfamily member 7 (TRPM7) is a permeable ion channel for divalent metal cations, preferentially permitting the flow of Mg^2+^ and Ca^2+^ (Nadler et al., 2001; Monteilh-Zoller et al., 2003; Penner and Fleig, 2007). TRPM7 has an essential role in the regulation of both cellular and whole body Mg^2+^ homeostasis, modulating fundamental cellular processes including cell division, growth, survival, differentiation, and migration (Ryazanova et al., 2010; Yee et al., 2014). The c-terminus of TRPM7 is a serine/threonine-protein kinase domain which functions as an intracellular sensor of magnesium status, and thus, provides coordination of cellular and systemic responses to magnesium deprivation (Ryazanova et al., 2014). In the whole body, TRPM7 is ubiquitously expressed and homozygous deletion of TRPM7 kinase domain is embryonic lethal, indicating that this molecule has a fundamental and non-redundant role in cellular physiology (Nadler et al., 2001; Ryazanova et al., 2010). Heterozygous KO mice for TRPM7 kinase domain (*Trpm7*^Δkinase∕+^ mice) are viable, but there is a change in magnesium homeostasis or hypomagnesemia-like phenotype. Sensitivity to intracellular Mg^2+^ levels is a critical mechanism to regulate the Mg^2+^ influx through TRPM7 channel into the cells, and the *Trpm7*^Δkinase∕+^ mice shows increased sensitivity to the inhibition by Mg^2+^ (Ryazanova et al., 2010, 2014).

Although, the magnesium deficiency is known to cause skeletal and tooth defects, the role of TRPM7 in hard tissue formation including the tooth mineralization has not been determined. Through, two whole transcript microarray analyses of varying stages of differentiating ameloblasts, we found that *Trpm7* was upregulated in secretory ameloblasts as compared to presecretory ameloblasts (GEO accession number GSE59214; Liu et al., 2015), and in maturation as compared to secretory ameloblasts (GEO accession number GSE57224; Zhang et al., 2014). Taking into consideration the significance of the maturation stage in enamel formation, where the majority of enamel mineralization occurs (Robinson et al., 1995; Smith, 1998), together with the importance of intracellular Mg^2+^ homeostasis in the skeletogenesis, we hypothesize that TRPM7 potentially contributes to the enamel matrix mineralization. In this study, we therefore confirmed the expression and synthesis of TRPM7 in differentiating ameloblasts, and further investigated the function of TRPM7 associated with the mineralization of craniofacial hard tissues using *Trpm7*^Δkinase∕+^ mice model. We determined a relationship of TRPM7 and tissue-nonspecific alkaline phosphatase activity to critically regulate the mineralization of craniofacial hard tissue.

## Methods

### Animals

All animal procedures were performed upon the approval by the Institutional Animal Care and Use Committee (IACUC) of the University of California, San Francisco and Rutgers Robert Wood Johnson Medical School, and adhered to the principles outlined in the National Institutes of Health Guide for the Care and Use of Laboratory Animals. *Trpm7*^Δkinase∕+^ and *wt* mice were provided by Dr. Alexey G. Ryazanov (Rutgers Robert Wood Johnson Medical School). As previously described, *Trpm7*^Δkinase∕+^ mice were genetically modified by replacing exons 32–36 of *Trpm7* gene, the kinase domain, with the Neo gene cassette (Ryazanova et al., 2010). At postnatal day 14 days, mice were euthanized, and whole heads were dissected out and fixed with 4% paraformaldehyde (PFA) overnight.

C57BL/6J female mice were maintained at the UCSF animal facility. At postnatal day 0 (P0), 5 (P5), and 10 (P10), mice were euthanized, developing first molars were harvested and processed for total RNA extraction. For immunohistochemical staining, 7-week old female C57BL/6J mice were anesthetized with 240 mg/kg tribromoethanol (Sigma-Aldrich, St. Louis, MO), fixed with 4% PFA for overnight.

### Total RNA extraction and semi-quantitative PCR (qPCR)

Total RNA was purified from developing molar tooth organ using the RNeasy Mini kit (Qiagen, Germantown, MD). The tooth organs were not homogenized therefore RNA would be primarily extracted from the exposed enamel epithelium overlying the tooth bud. cDNA was obtained by reverse transcription of the mRNA using Superscript III First-Strand Synthesis Supermix for qRT-PCR (Life Technologies, Grand Island, NY).

Expression of *Trpm7* was examined by semi-quantitative PCR with FastStart Universal SYBR Green Master Kit (Roche Diagnostics, Indianapolis, IN) using the ABI 7500 system (Applied Biosystems, Foster City, CA). The primer sequences for *Trpm7* are: sense 5′-ATGGCACTGTTG GAAAGTATGG-3′, antisense 5′-CGCCTTCAA ATATCAAAGCCAC-3′; *Eef1a1* was used as a reference gene, the primer sequences are: sense 5′-CAA CAT CGT CGT AAT CGG ACA-3′, antisense 5′-GTC TAA GAC CCA GGC GTA CTT-3′. The expression levels of target gene was analyzed using the ΔΔCt method (Livak and Schmittgen, 2001). The relative expression levels of *Trpm7* of P5 and P10 enamel organs were calculated based on the expression levels of P0 enamel organs. Significance of differences was determined using ΔCT values by the multiple *t*-test with Bonferroni correction following ANOVA (Baker et al., 2006; Yuan et al., 2006).

### Micro-computed tomography (MICROCT)

Whole heads from *wt* and *Trpm7*^Δkinase∕+^ mice were fixed in 4% PFA overnight, and then imaged using a Micro XCT-200 system (Xradia, Pleasanton, CA). All scans were done at an operating voltage of 90 KVp and 66 μA of current, at an optical magnification 2x. A binning of 2 was used for 3D image reconstruction. All scans were done using the same experimental settings, including the distances between specimen, detector, and source. Virtual sections were converted to bmp images using the Xradia TXM3DViewer 1.1.6. software. Appropriate imaging planes were selected from three orthogonal sections centered at a level containing an entire sagittal slice of the incisor or entire frontal slice of the first molar containing the mesial and buccal cusps and mesial root inside the reconstructed space using Xradia TXM3DViewer 1.1.6. software.

### Immunohistochemistry

The mandibles and maxillae were decalcified in 8% EDTA (pH 7.3) at 4⋅C for 2 wks (7-week-old C57BL/6J mice) or 1 wk (*wt* and *Trpm7*^Δkinase∕+^ mice). The jaws were further dehydrated through a graded series of ethanol, followed by a routine paraffin embedding. The paraffin blocks were sectioned at the thickness of 5 μm. After dewaxing, the sections used for TRPM7 immunostaining were subjected to the antigen retrieval in 1% SDS in 0.1 M Tris-HCL buffer (pH 9.0) for 5 mins at room temperature (Brown et al., 1996; Emoto et al., 2005), and then treated with 1% H_2_O_2_ for 5 min at room temperature. Afterward, all sections were incubated with the blocking reagent containing 10% swine and 5% goat sera for 2 h at room temperature followed by an incubation with either rabbit anti-TRPM7 antibody (Abcam, Cambridge, United Kingdom) overnight at 4⋅C or rabbit anti-human ALPL (Abcam) antibody overnight at 4⋅C. Sections were further incubated with biotin conjugated swine anti-rabbit F(ab')_2_ secondary antibody (Dako Denmark A/S, Glostrup, Denmark) for 1 h at room temperature. Next, the sections incubated with anti-TRPM7 antibody were incubated with intestinal alkaline phosphatase (ALPase) conjugated streptavidin (Vector Laboratories Inc., Burlingame, CA) for 30 mins, and immunoreactivity was visualized using a Vector® Red kit (Vector Laboratories Inc.) resulting in pink/red color for positive staining. To block the endogenous tissue-nonspecific ALPase activity, 1 mM levamisole was added to the visualization reagent. The sections incubated with anti-ALPL antibody were incubated with horseradish peroxidase (HRP) conjugated streptavidin (Vector Laboratories Inc.) for 30 mins, and immunoreactivity was visualized using an ImmPACT tM DAB peroxidase substrate kit (Vector Laboratories Inc.), resulting in dark brown color for positive staining. Nuclear counterstaining was performed with methyl green (Dako Denmark A/S). Normal rabbit IgG was used as the negative control.

### Histological analysis

The fixed mandibles and maxillae were dehydrated in acetone, and embedded in Technovit 8100 resin (Heraeus Kulzer GmbH, Hanau, Germany). Undecalcified sections were obtained at the thickness of 3 μm. Mineralization of the tissue was visualized by 2.5% silver nitrate staining (von Kossa stain). Bone and dentin mineralization was further assessed by following Goldner's Trichrome stain protocol (Goldner, 1938). For general morphological analysis, sections were stained with 1% toluidine blue.

### Enzyme histochemistry for ALPase activity

To detect ALPase activity, the undecalcified Technovit sections were incubated with the modified Burstone's reagent comprising 1.5 mM Naphthol AS-MX phosphate, 0.5 mM Fast Red Violet LB salt and 3 mM MgSO_4_ in 0.1 M Tris–HCl buffer (pH 9.2) at 37⋅C for 60 mins (Nakano et al., 2004). Some of the sections were pre-incubated with 0.1 M Tris–HCl buffer (pH 7.3) supplemented with 50 mM MgSO_4_ for 1 day at 4⋅C (Yoshiki et al., 1972; Nakano et al., 2004).

## Results

### Expression and synthesis of Trpm7 progressively increased with ameloblast differentiation

**Figure 1:**
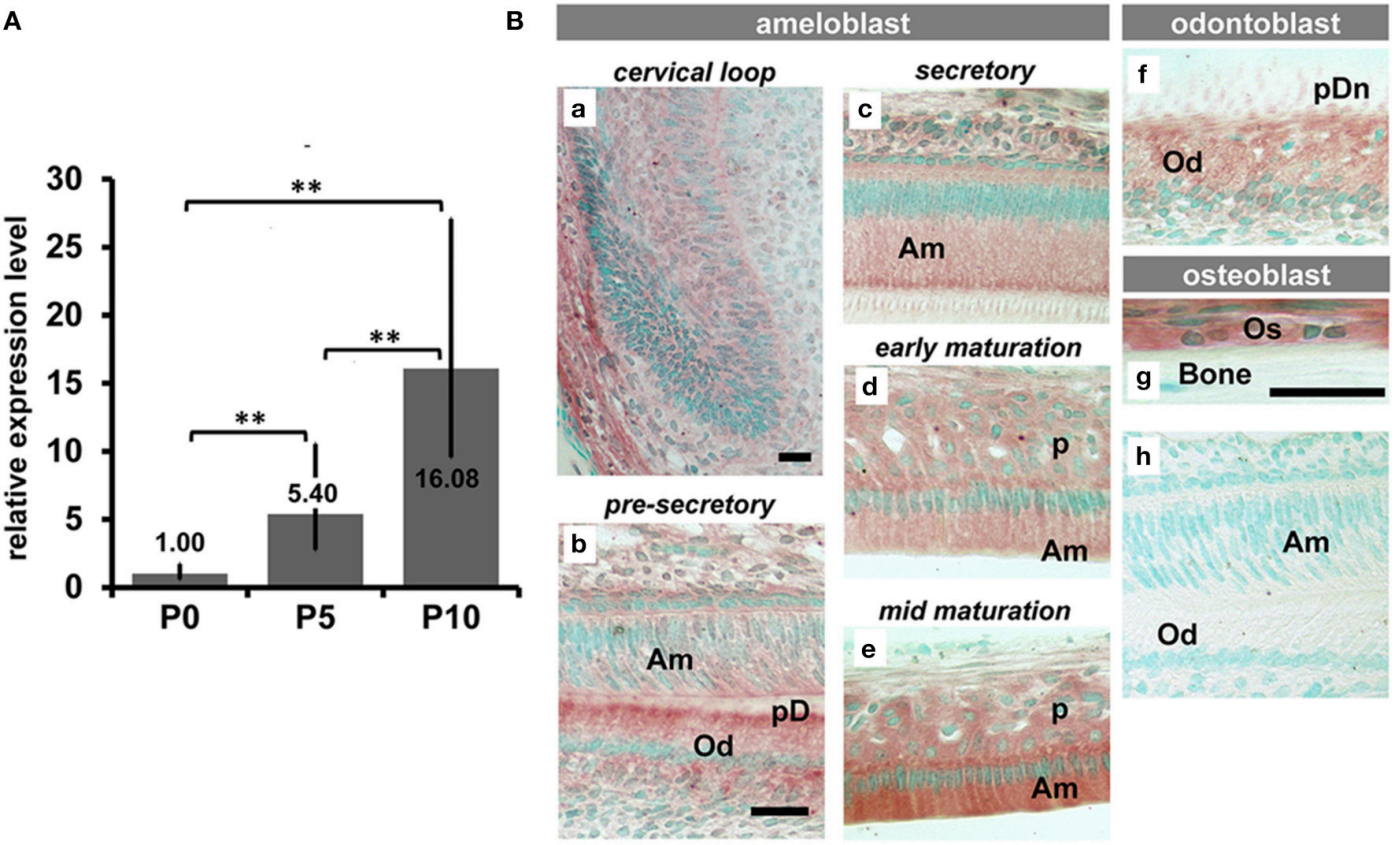
**The expression pattern of TRPM7 in craniofacial hard tissue forming cells. (A)** Semi quantitive PCR analysis showed that the expression levels of TRPM7 mRNA in mouse molar enamel organ progressively increased from pre-secretoy stage (P0) to secretory stage (P5) and to early maturation stage (P10) (^**^P < 0.01). **(B)** Immunohistochemical staining for TRPM7 on incisors and surrounding alveolar bone showed an increased intensity of TRPM7 as ameloblasts progressed from precursor cells at cervical loop to maturation stage **(Ba–e)**. TRPM7 was also immunolocalized in odontoblasts **(Bb,f)**, and osteoblasts **(Bg)**. No immunoreactions were seen in those cells immunostained with non-specific rabbit IgG **(Bh)**. Scale bars: 25 μm

Analysis of our previous microarray data (GSE59214 and GSE57224) revealed that expression of *Trpm7* was progressively upregulated from pre-secretory ameloblasts, to secretory ameloblasts, then to maturation ameloblasts. To confirm this, we used qPCR to compare relative *Trpm7* expression in the ameloblasts obtained from developing mouse enamel organs at three differentiation stages; pre-secretory/P0, secretory/P5 and early maturation/P10 stages. The qPCR analysis showed that the relative expression level of *Trpm7* compared with pre-secretory ameloblasts was 5.4-fold in secretory ameloblasts and 16.08-fold in maturation stage ameloblasts (Figure 1A). TRPM7 protein was detected in ameloblasts of all stages, and the intensity of the immunostaining increased as differentiation of ameloblasts advanced, with the highest immunostaining signal in maturation ameloblasts (Figures 1Ba–e). Moreover, we found that the odontoblasts (Od) and osteoblasts (Os) were also immunostained for TRPM7 (Figures 1Bb,f,g). No immunoreaction was detected on the negative control sections (Figure 1Bh).

### Significantly hypomineralized enamel, dentin and cranial bones are found in *Trpm7*^Δkinase∕+^ mice

**Figure 2:**
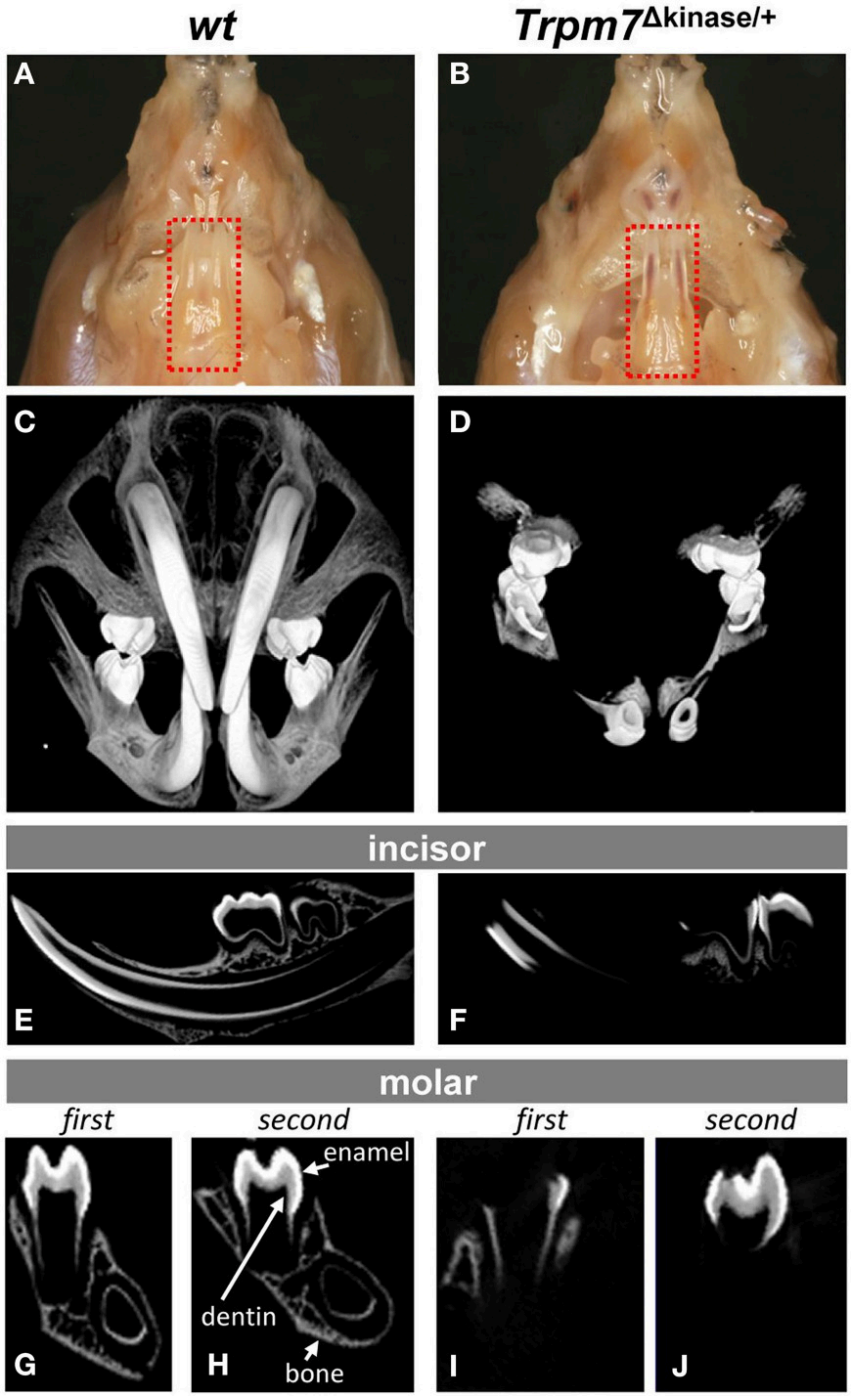
**A hypomineralized phenotype is present in enamel, dentin and cranial bones of *Trpm7*^Δkinase∕+^ mice**. **(A)** Transluscent enamel is found in P14 *wt* mice. **(B)** The transparent mandibular incisor enamel of P14 *Trpm7*^Δkinase∕+^ mice allows the red colored pulp tissue could be seen through. **(C)** 3-D microCT images of craniofacial structure show well mineralized incisors, entire molars, and craniofacial bones in *wt* mice. **(D)** At the same intensity threshold, the mineralized tissues are only detected in the crowns of molars, a small segment of incisors near the incisal end in *Trpm7*^Δkinase∕+^**. **(E)** A representive 2-D microCT image of sagittal section from P14 *wt* mouse hemimandible show the well contrasted enamel, dentin and alveolar bone. **(F)** The 2-D microCT image of sagittal section from P14 *Trpm7*^Δkinase∕+^ hemimandibles show contrasting image intensity consistent with mineralization only seen in the molar crown and incisal end of the incisor. Mineralized enamel, dentin and alveolar bone are detected in P14 *wt* first molar **(G)**, and second molar **(H)**. Clear contrasts in these tissues is only seen in part of first molar enamel, dentin and alveolar bone **(I)** and coronal enamel and dentin in the second molar **(J)** of P14 *Trpm7*^Δkinase∕+^ hemimandible.

To further understand the TRPM7 functions in enamel and dentin formation, and craniofacial skeletogenesis, we compared the craniofacial structure of *Trpm7*^Δkinase∕+^ *mice* to *wt* controls. Incisors of P14 *wt* mice were hard and translucent (Figure 2A), whereas *Trpm7*^Δkinase∕+^ mice were soft, and the red vasculature enriched dental pulp was easily seen through the enamel and dentin layers (red box in Figure 2B). Moreover, craniofacial bones were also soft and easily bent like a piece of paper.

Consistent with the gross morphology, the 3D microCT of craniofacial structure showed that in *wt* mice, the well mineralized incisors, molars, and craniofacial bones were distinguishable at the same intensity (Figure 2C), while in *Trpm7*^Δkinase∕+^ mice, mineralization was only detected in the crowns of molars, and in smaller area of the incisors specifically at incisal end (Figure 2D). Virtual sagittal sections from the *Trpm7*^Δkinase∕+^ hemimandibles further demonstrated limited mineralization in the crown of the molar and the incisor (Figures 2F,I,J) as compared to *wt* controls (Figures 2E,G,H).

**Figure 3:**
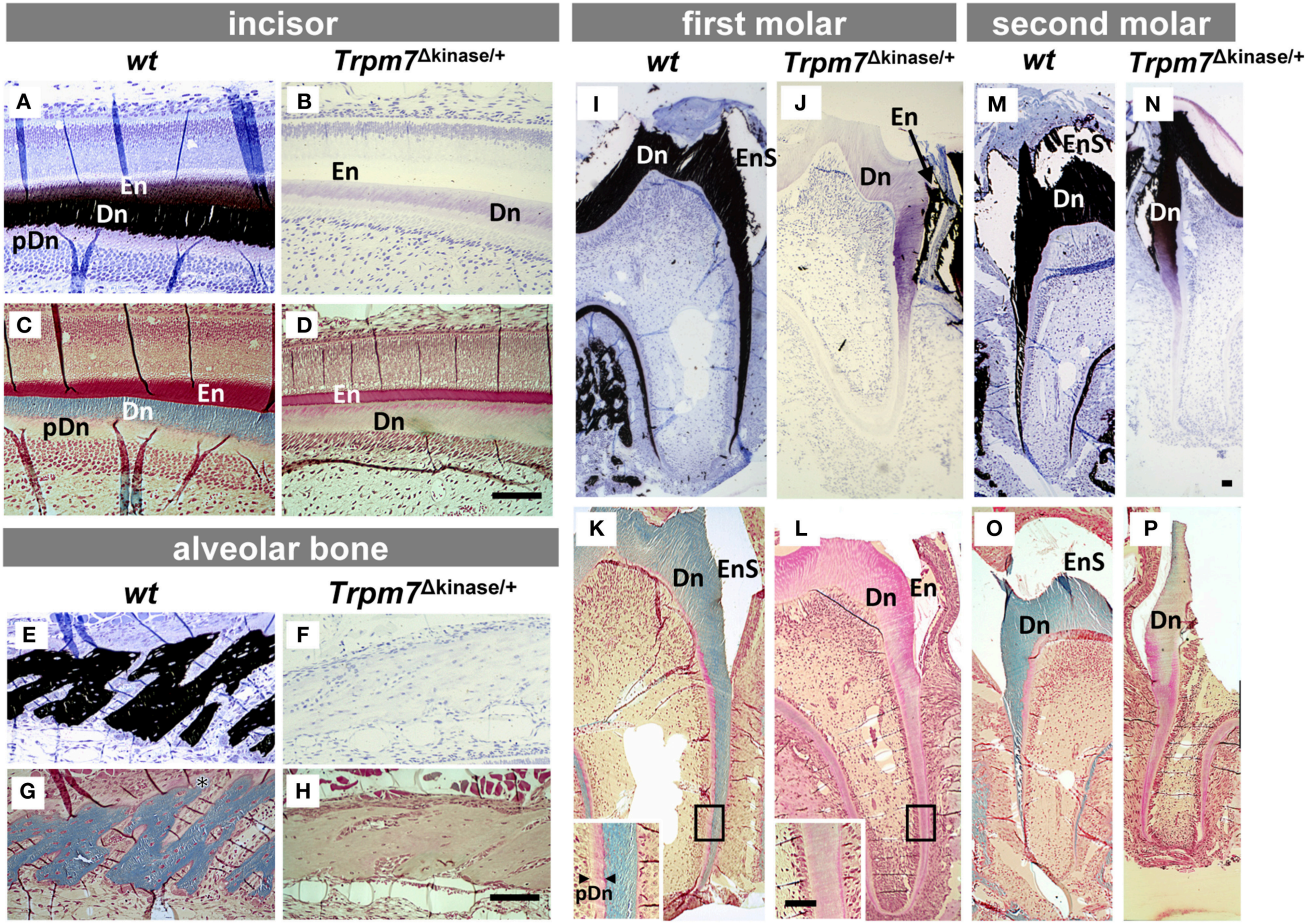
**Hypomineralization defects of enamel, dentin and alveolar bone in *Trpm7*^Δkinase∕+^ mice are characterized by histological analyses**. Matrix mineralization was assessed by Von Kossa staining **(A,B,E,F,I,J,M,N)** and Goldner's Trichrome staining **(C,D,G,H,K,L,O,P)**. In the *wt* incisor **(A**,**C)**, secretory stage enamel matrix (En) is lighty positive (black) for Von Kossa staining **(A)**, and is stained in red by Trichrome staining **(C)**. Dentin matrix (Dn) is stained in black with Von Kossa **(A)**, and is stained in blue with Trichrome staining **(C)**. Non-mineralized/Von Kossa negative pre-dentin (pDn) is stained in light pink by Trichrome staining **(C)**. In *Trpm7*^Δkinase∕+^ mice, both incisal enamel and dentin are negative for Von Kossa staining **(B)**, and Trichrome stains the dentin matrix in light pink, similar to the color of non-mineralized pre-dentin **(D)**. The alveolar bone matrix of *wt* mice is stained in black by Von Kossa **(E)**, and light blue by Trichrome staining **(G)**. The alveolar bone matrix of *Trpm7*^Δkinase∕+^ mice is negative for Von Kossa staining **(F)**, and Trichrome staining on alveolar bone showed bone in *Trpm7*^Δkinase∕+^ mice is light pink **(H)**, similar to osteoid. Unlike Von Kossa positive stained *wt* molar dentin **(I,M)**, the entire dentin of the *Trpm7*^Δkinase∕+^ first molar is negative for Von Kossa staining **(J)** and shows pink/pre-dentin status by Trichrome staining **(L)**. Interestingly, in the second molar of *Trpm7*^Δkinase∕+^ mice, both Von Kossa **(N)** and Trichrome **(P)** staining shows the coronal dentin as partially mineralized, but the root dentin is not mineralized. Enamel matrix of molars likely chipped off during the sectioning (seen as an enamel space/EnS), and the remaining matrix is positive for Von Kossa staining **(N)**. Scale bars: 100 μm

Histological assessment on the undecalcified mandibular sections showed von Kossa positive stained (in dark) enamel (En), dentin (Dn), and alveolar bone (Figures 3A,E,I,M), and a lack of von Kossa positive staining on the *Trpm7*^Δkinase∕+^ incisor enamel and dentin, molar root and alveolar bone (Figures 3B,F,J,N). Trichrome staining showed that dentin and bone in the *wt* mice were stained in blue (Figures 3C,G,K,O), while incisor dentin, molar root dentin and bones in *Trpm7*^Δkinase∕+^ mice were stained in pink, although the width of dentin and bone layer was comparable (Figures 3D,H,L,P), indicating that the dentin and bone matrix in the *Trpm7*^Δkinase∕+^ mice was deposited at the same levels as *wt* mice but the mineralization did not proceed normally. The coronal dentin of the first molars of *Trpm7*^Δkinase∕+^ mice was negative for von Kossa staining (Figure 3J), while the coronal dentin of the second molar was positive for the same staining (Figure 3N). Consistently, by staining with Trichrome, the mineralization status of the coronal dentin was also confirmed as pink/unmineralized, in the first molars (Figure 3L), and blue gray/mineralized, in the second molar (Figure 3P).

### Ameloblast morphology is not significantly altered in *Trpm7*^Δkinase∕+^ mice

**Figure 4:**
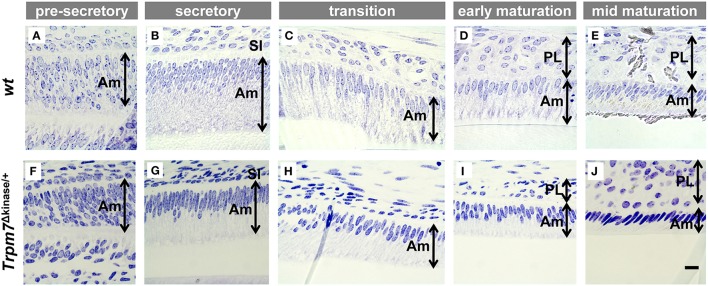
**There are no obvious morphological change in ameloblasts of *Trpm7*^Δkinase∕+^ mice**. Morphodifferentiation of ameloblasts (Am) from pre-secretory stage to secretory stage in *Trpm7*^Δkinase∕+^ mice **(F–J)** is similar to that of *wt* mice **(A–E)**. Am, ameloblast; SI, stratum intermedium; PL, papillary layer; scale bars: 25 μm

The morphology of ameloblasts is associated with the functional property of each differentiation stage. As mentioned above, TRPM7 was immunolocalized in ameloblasts in a stage-specific manner. Therefore, we examined the morphology of ameloblasts by Toluidine blue staining in order to determine whether TRPM7 is critical for the morphological differentiation of ameloblasts. The morphological property of the ameloblasts in the *Trpm7*^Δkinase∕+^ mice at all stages remained similar to that of the *wt* mice throughout the differentiation. Only the height of ameloblast layer appeared to be slightly shorter (Figure 4).

### Acellular cementum is absent in *Trpm7*^Δkinase∕+^ mice

**Figure 5:**
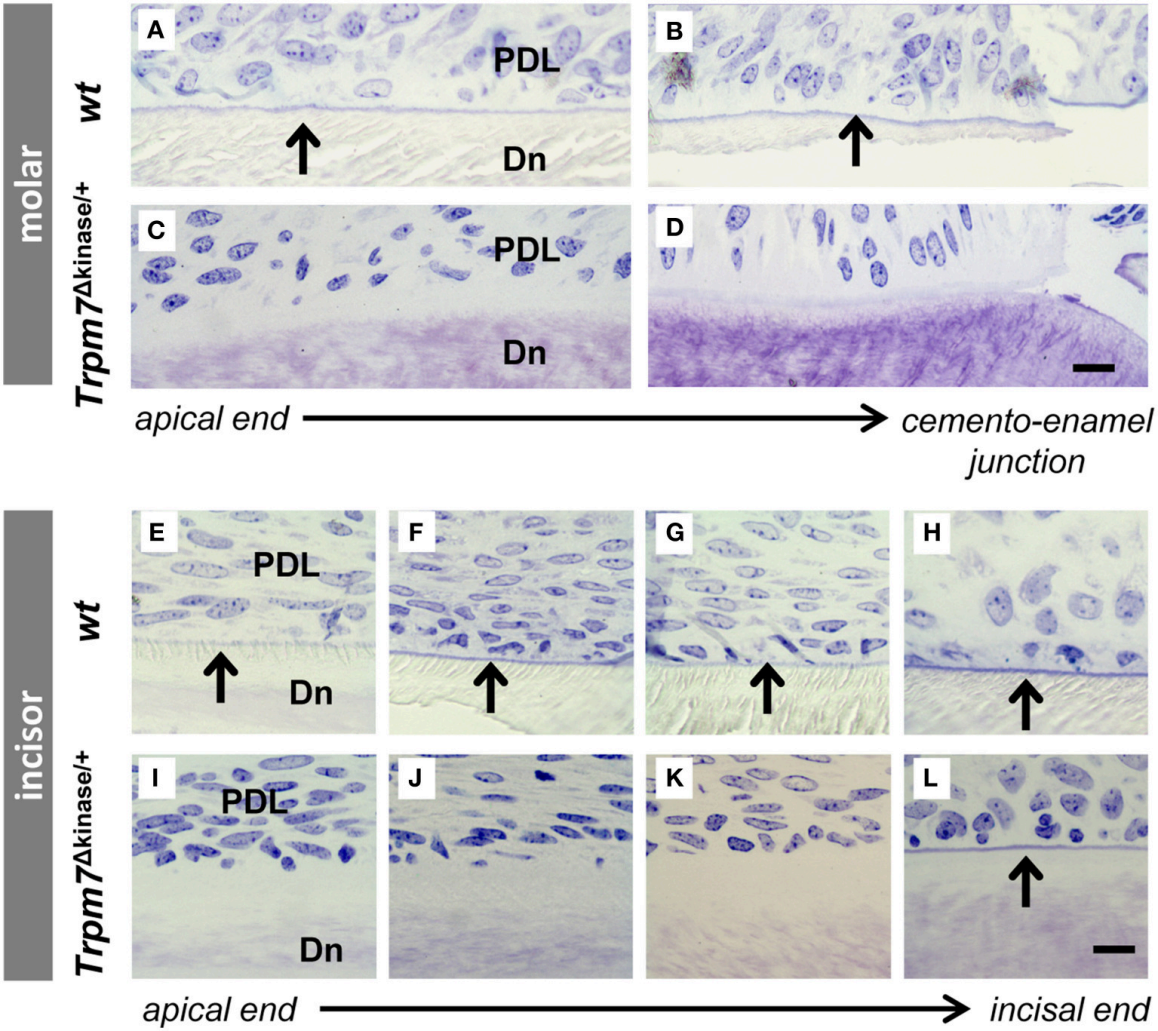
**Acellular cementum is found on the root analog of *wt* mouse incisor as well as on the molar root**. Acellular cementum, ususally seen as a blue line following toluidine blue staining, is visualized between periodontal ligment (PDL) and dentin (Dn) on *wt* molar **(A,B)**. A similar staining pattern corresponding to acellular cementum can not be detected in *Trpm7*^Δkinase∕+^ mice **(C,D)**. Acellular cementum, seen as a blue line by Toluidine Blue staining, is visualized along the incisors from the apical to incisal end in P14 *wt* mice (**E–H**, indicated by black arrows). However, this line (indicated by black arrow) only appears on the surface of the incisor root analog dentin near the incisal end in *Trpm7*^Δkinase∕+^ mice **(I–L)**. Scale bars: 25 μm

Through further morphological studies, we found that acellular cementum in molars, normally stained as a deep blue line by toluidine blue in undecalcified sections (McKee et al., 2011; Figures 5A,B), was absent in the *Trpm7*^Δkinase∕+^ mice (Figures 5C,D). While the most of the root analog surface was covered by the acellular cementum in *wt* incisors (Figures 5E–H), acellular cementum was only detected near the incisal end on the incisor of *Trpm7*^Δkinase∕+^ mice (Figures 5I–L).

### *Ex vivo* ALPase activity is abolished in the *Trpm7*^Δkinase∕+^ mice, but can be rescued in sections pre-incubated with Mg^2+^ solution

**Figure 6:**
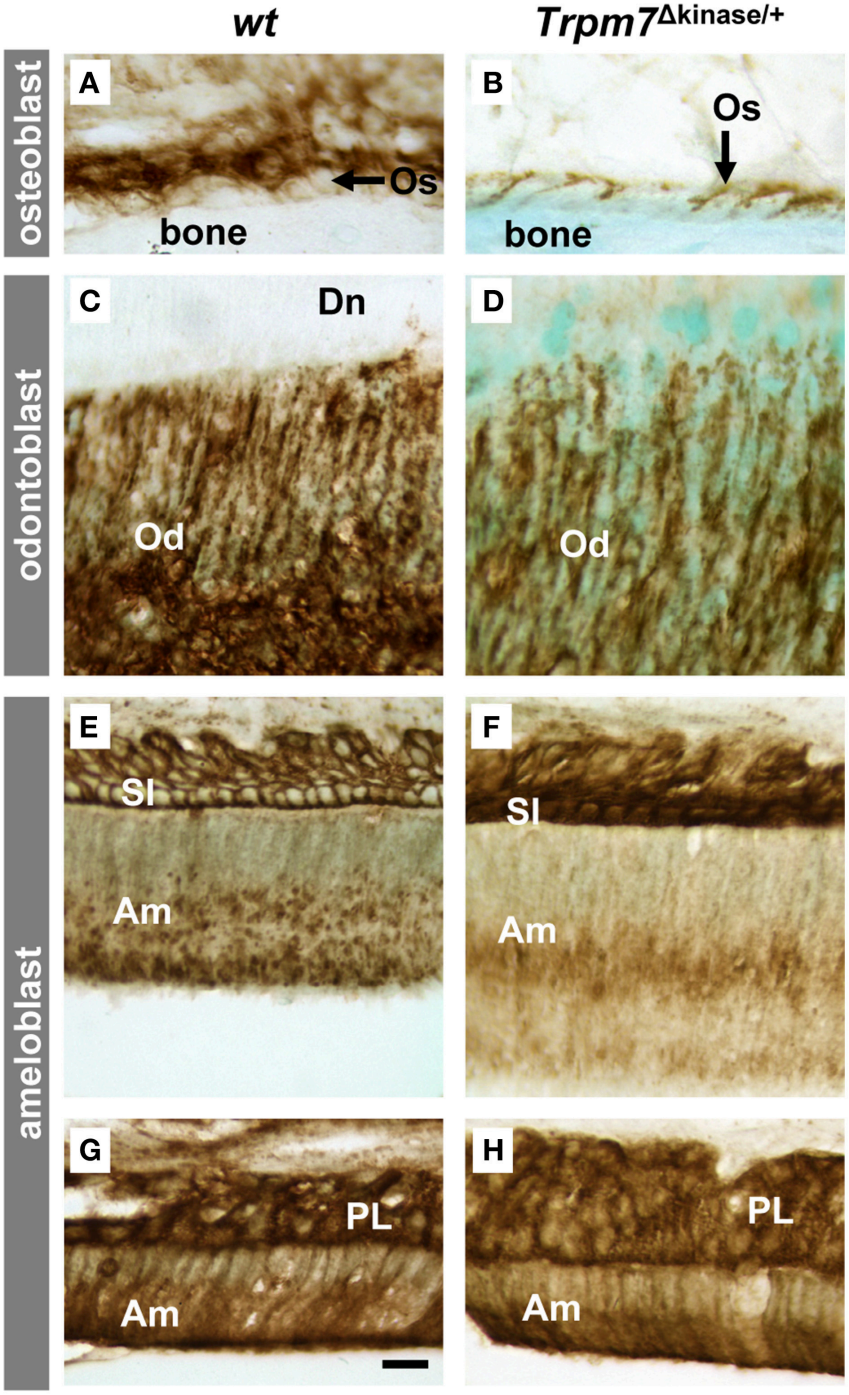
***Trpm7* gene deficiency does not affect the abundance of ALPL protein**. In both *wt* and *Trpm7*^Δkinase∕+^ mice, ALPL was immunolocalized on the basal and lateral plasma membrane of osteoblasts (Os) **(A,B)** and odontoblasts (Od) **(C,D)** at the similar levels. In enamel organs of both *wt* and *Trpm7*^Δkinase∕+^ mice, ALPL was immunolocalized on plasma membrane of stratum intermedium cells (SI) and the cytoplasm of ameloblasts (Am) at secretory stage **(E,F)**, and on the plasma membrane and cytoplasm of papillary layer cells (PL) and maturation ameloblasts **(G,H)**. There was no notable difference in immunoreactive activities between *wt* and *Trpm7*^Δkinase∕+^ mice. Scale bars: 25 μm

**Figure 7:**
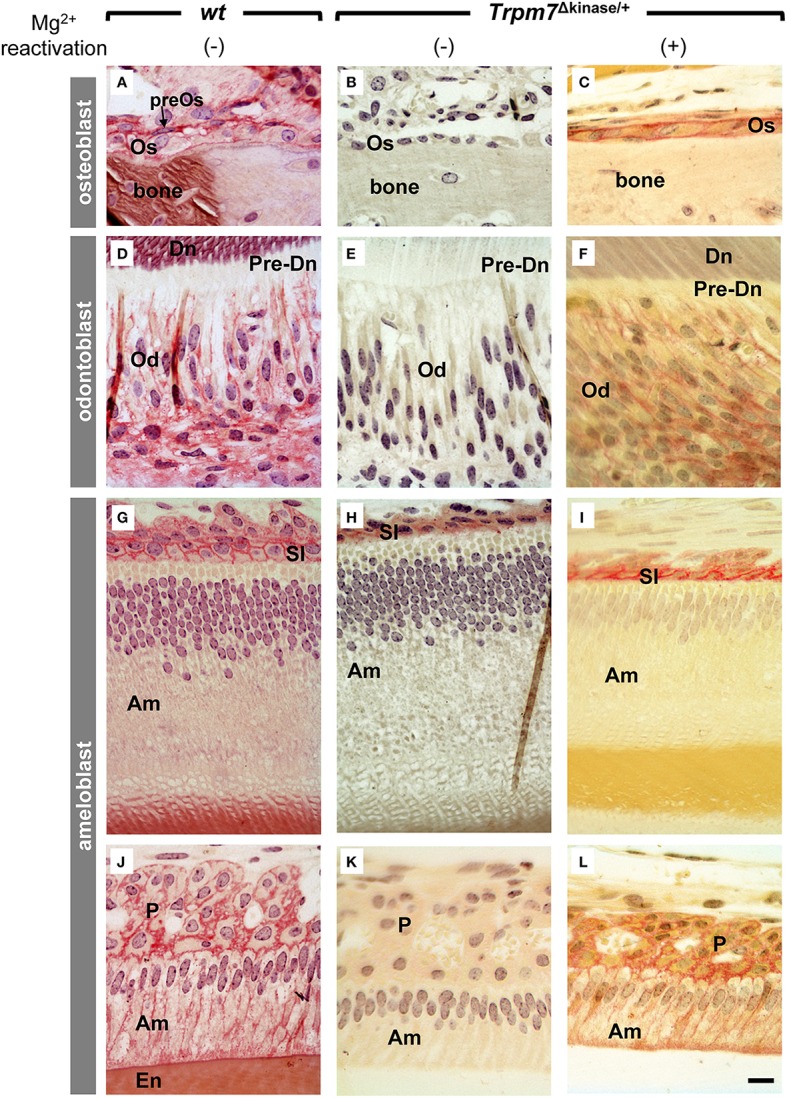
**Impaired alkaline phosphatase (ALPase) activity of osteoblasts, odotoblasts and ameloblasts in *Trpm7*^Δkinase∕+^ mice is partially retrieved by Mg^2+^ pre-treatment**. **(A)** At the bone forming site, ALPase activity was detected (in red) on the plasma membrane of osteoblasts (Os) and its precursor cells (preOs) in *wt* mice. **(B)**There was no ALPase activity presented by osteoblasts in *Trpm7*^Δkinase∕+^ mice. **(C)** However, Mg^2+^ pre-treatment retrieved the ALPase activity on osteoblasts. **(D)** ALPase activity was detected at the basolateral surface of odontoblasts (Od) in *wt* mice. **(E)** While in *Trpm7*^Δkinase∕+^ mice, no ALPase activity was seen in the odontoblasts. **(F)** Mg^2+^ pre-treatment retrieved ALPase activity on odontoblasts. **(J)** ALPase activity was detected in stratum intermedium (SI), secretory ameloblasts (Am), and **(G)** papillary layer cells (P) at maturation stage in *wt* mice. In *Trpm7*^Δkinase∕+^ mice, ALPase activities were restricted only on the stratum intermedium at secretory stage, not in ameloblasts **(H,K)**. Mg^2+^ pre-treatment retrieved the ALPase activity on ameloblasts and papillary layer cells **(I,L)**. Dn, dentin; pre-Dn, pre-dentin; En, enamel; Scale bars: 25 μm.

We found that the mineralization phenotypes in *the Trpm7*^Δkinase∕+^ *mice*, including hypomineralization in bone, incisor and molar root and absence of acellular cementum, resembled those of the tissue-nonspecific alkaline phosphatase (*Alpl*) KO mice (Beertsen et al., 1999; McKee et al., 2011). These phenotypes led us to examine the abundance of ALPL and *ex vivo* ALPase enzyme activity in osteoblasts (Os), odontoblasts (Od), and ameloblasts (Am). The intensity and localization of ALPL immunostaining in Os, Od, and enamel organ cells, including Am, stratum intermedium (SI) and papillary layer cells (PL), was similar in *wt* and *Trpm7*^Δkinase∕+^ *mice* (Figure 6). However, ALPase activity present in *wt* mice (Figures 7A,D,G,J) was absent in the *Trpm7*^Δkinase∕+^ mouse, except the stratum intermedium cells (Figures 7B,E,H,K). Nevertheless, the *ex vivo* ALPase activity was largely rescued in sections of *Trpm7*^Δkinase∕+^ mineralizing tissue by the pre-incubation of the sections with MgSO_4_ solution prior to staining for ALPase activity (Figures 7C,F,I,L).

## Discussion

Tooth enamel is the hardest mineralized tissue in the vertebrate body comprised of 92 volume% inorganic mineral, which is higher than those in dentin and bones (61 and 64.5 volume% respectively; Kumar, 2014). Such a high mineralization rate of enamel is primarily achieved by maturation ameloblasts, which are able to efficiently remove the hydrolyzed enamel matrix proteins and deposit minerals into enamel matrix during the final phase of mineralization. We found that transient receptor potential cation channel, subfamily M, member 7 (TRPM7) was significantly upregulated in maturation ameloblasts. Characterization of craniofacial structures of mice *Trpm7*^Δkinase∕+^ mice, showed a severe hypomineralized phenotype in all mineralized tissues, indicating a universal role of TRPM7 in tissue mineralization.

Although, some unique molecular mechanisms are employed during enamel mineralization, there are many common factors that regulate the mineralization of enamel, dentin and bone. For instance, tissue-nonspecific alkaline phosphatase (ALPL) is known to be a key regulator of bone and tooth mineralization. Mice lacking *Alpl* show defects in bone, dentin and enamel mineralization and absence of acellular cementum (Waymire et al., 1995; Narisawa et al., 2001; McKee et al., 2011). In humans, mutations in *ALPL* genes cause hypophosphatasia, a rare inherited disorder characterized by deficiency of serum and bone alkaline phosphatase activity, resulting in the defective bone and tooth mineralization (Mornet, 2007). Physiological functions of ALPL are not fully understood yet, nevertheless, in bone and probably dentin, where the mineralization occurs in the type 1 collagen dominant matrix, ALPL is shown to initiate and direct mineralization by removing pyrophosphate (PPi), a mineralization inhibitor, (Addison et al., 2007) and antagonizing generation of PPi (Johnson et al., 2000; Hessle et al., 2002). Though ALPL has been shown to be present in secretory and maturation stage of ameloblasts (Bevelander and Johnson, 1949; Gomez and Boyde, 1994), the significance of ALPL in enamel formation has not been understood yet.

Divalent metal ions, including Mg^2+^, Zn^2+^, and Ca^2+^, are essential for ALPase activity (Stec et al., 2000; Hoylaerts et al., 2015). Magnesium deficient rats showed significantly reduced plasma ALPase activity, which was partially restored by an *in vitro* magnesium supplementation (Heaton, 1965). TRPM family, which consists of eight members, plays an essential role for magnesium entering the cells (Ryazanova et al., 2014). In analysis of our previously published microarray data (Zhang et al., 2014; Liu et al., 2015), we identified that TRPM7 was the only gene, among the well-known magnesium transporters (such as TRPM6, MAGT1, MRS2, PPM1G, MMGT1, and NIPAL1), which was significantly upregulated in ameloblasts as compared to other epithelial cells.

*Trpm7*^Δkinase∕+^ mice are known to have significantly lower plasma, bone and urine magnesium levels as compared with *wt* mice, and also show behavioral defects (clasping, tremors, and seizures) and other hypomagnesemia-like phenotype (Ryazanova et al., 2010). Interestingly, a seizure is also one of the behavioral phenotypes displayed by *Alpl* KO mice, and has been linked to a lack of vitamin B6 metabolized by ALPL (Waymire et al., 1995; Whyte et al., 1995; Narisawa et al., 1997; Mackey et al., 2006). Magnesium deficiency is shown to impair vitamin B6 status by inhibiting plasma ALPase activity in rat (Planells et al., 1997), suggesting that the activity of tissue non-specific ALPase (ALPL) is repressed under hypomagnesaemia condition.

Our microCT and morphological analyses on the hemimandibles of *Trpm7*^Δkinase∕+^ mice revealed similarity in skeletal and dental phenotypes, including partially mineralized molar crowns and absence of acellular cementum, between *Alpl* KO mice (Beertsen et al., 1999; McKee et al., 2011) and *Trpm7*^Δkinase∕+^ mice. These observations suggested a possibility that ALPL in the *Trpm7*^Δkinase∕+^ mice might be affected. In support of this possibility, we found that although *Alpl* protein content was similar in *wt* and *Trpm7*^Δkinase∕+^ mouse ameloblasts, odontoblasts and osteoblasts, *ex vivo ALPase* activity was dramatically reduced in *Trpm7*^Δkinase∕+^ *mice*. Restoration of *ex vivo* ALPase activity by Mg^2+^ pre-incubation of sections prior to staining for ALP activity, indicates that magnesium deficiency is a major cause for the deficient ALPase activity of *Trpm7*^Δkinase∕+^ mice. It is worth noting that there are also numerous enzymes that require Mg^2+^ for their activities. Therefore, hypomineralized tooth and bone in the *Trpm7*^Δkinase∕+^ mice could be also due to the cumulative deficiency of some other enzymes as well.

Nevertheless, in this study, the *ex vivo* supplementation of magnesium could not completely restore the ALPase activity to the levels displayed in the *wt* cells. This phenomenon is similar to what is found in *ex vivo* ALPase activity in tissue sections from Mg^2+^ deficient rats (Heaton, 1965). The TRPM7 channel is also permeable to Ca^2+^, and therefore the lack of TRPM7 could also reduce the amount of available intracellular calcium. Optimized Ca^2+^ is critical for ALPase activity (Hoylaerts et al., 2015), and therefore, although Mg^2+^ concentrations are restored, reduced calcium concentrations could impact ALPase activity. In addition, magnesium deficiency is known to have a secondary effect on the metabolism of Ca^2+^, K^+^ and inorganic phosphate (Konrad et al., 2004; Rude and Shils, 2006), therefore, the hypomineralization of *Trpm7*^Δkinase∕+^ mice was also attributed to an altered intra and/or extra cellular Ca^2+^ and inorganic phosphate.

In this study, we found that TRPM7 was highly expressed by ameloblasts, odontoblasts and osteoblasts, which are cells responsible for the formation of enamel, dentin and bone respectively. All the mineralized tissues in the TRPM7 kinase domain deficient mice are hypomineralized, with reduced ALPase activity. Unlike bone and dentin, where ALPL functions in the initial phase of mineralization, in enamel formation, it is thought that ALPL functions at the maturation stage, where the final but majority of mineralization takes place (Takano and Ozawa, 1980). Our finding that ubiquitous inhibition of mineralization in all stage of enamel formation suggested that the initiation of enamel matrix mineralization at the secretory stage is also regulated by ALPL. In support of this, our immunohistochemistry showed that ALPL was apparently present in the cytoplasm of secretory ameloblasts. Therefore, similar to bone and dentin, ALPL also possibly contributes to the initiation of enamel matrix mineralization at the secretory stage by fine-tuning the local concentration of PPi.

Of course, there are also numerous enzymes that require Mg^2+^ for their activities. Therefore, hypomineralized tooth and bone in the *Trpm7*^Δkinase∕+^ mice could be also due to the cumulative deficiency of some other enzymes as well.

In conclusion, our findings suggest that TRPM7 plays a critical role in the mineralization of enamel as well as dentin and craniofacial bone via regulating activity of ALPL by transporting Mg^2+^, which is necessary for ALPL activity, into the cells.

## Author contributions

All authors listed, have made substantial, direct and intellectual contribution to the work, and approved it for publication.

### Conflict of interest statement

The authors declare that the research was conducted in the absence of any commercial or financial relationships that could be construed as a potential conflict of interest.
